# Hypoglycemia in Children: Major Endocrine-Metabolic Causes and Novel Therapeutic Perspectives

**DOI:** 10.3390/nu15163544

**Published:** 2023-08-11

**Authors:** Alessia Quarta, Daniela Iannucci, Miriana Guarino, Annalisa Blasetti, Francesco Chiarelli

**Affiliations:** Department of Pediatrics, University of Chieti—Pescara, Gabriele D’Annunzio, 66100 Chieti, Italy; alessiaquarta54@gmail.com (A.Q.); daniela.iannucci.88@gmail.com (D.I.); mirianag_@hotmail.it (M.G.); ablasetti@tiscali.it (A.B.)

**Keywords:** hypoglycemia, glucose homeostasis, endocrine hypoglycemia, inborn errors of metabolism

## Abstract

Hypoglycemia is due to defects in the metabolic systems involved in the transition from the fed to the fasting state or in the hormone control of these systems. In children, hypoglycemia is considered a metabolic-endocrine emergency, because it may lead to brain injury, permanent neurological sequelae and, in rare cases, death. Symptoms are nonspecific, particularly in infants and young children. Diagnosis is based on laboratory investigations during a hypoglycemic event, but it may also require biochemical tests between episodes, dynamic endocrine tests and molecular genetics. This narrative review presents the age-related definitions of hypoglycemia, its pathophysiology and main causes, and discusses the current diagnostic and modern therapeutic approaches.

## 1. Introduction

Definition of hypoglycemia remains controversial in children [1]. Clinically, hypoglycemia is defined as a plasma glucose concentration low enough to cause signs and symptoms of brain dysfunction or neuroglycopenia [2,3]. However, it is difficult to identify a single glucose value below which this symptomatology appears, which is influenced by multiple factors, particularly the availability of alternative energy substrates for the brain such as ketone bodies, as well as severity, duration and recurrence of hypoglycemic episodes. According to the American Academy of Pediatrics (AAP) and Pediatric Endocrine Society (PES), hypoglycemia is diagnosed when plasma glucose is, respectively, <47 mg/dL and <50 mg/dL in at term newborns during the first 48 h of life [2]. Different threshold values have been proposed for pre-term infants [4]. In at-term newborns after the first 48 h of life, infants and younger children, hypoglycemia is defined when plasma glucose is <50 mg/dL [5]. This threshold value is low enough to avoid false-positive results, but is unlikely to lead to permanent neurological damage [6,7,8]. In older children, it is possible to use Whipple’s triad characterized by signs and/or symptoms of hypoglycemia, reduced plasma glucose concentration and resolution of these signs/symptoms after acquisition of normoglycemic status [9].

### 1.1. Pathophysiology

**Figure 1:**
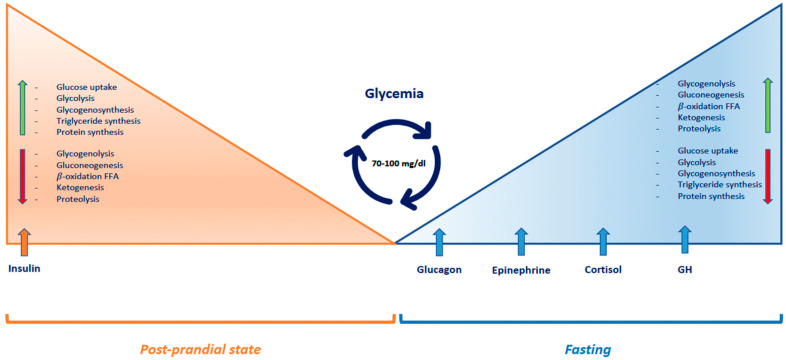
Regulatory mechanisms of glucose homeostasis. Abbreviations: FFA: free fatty acids, GH: growth hormone.

Glucose is the main energy substrate for the brain. Since the intracerebral transport of glucose depends on its plasma concentration and the brain can store only limited reserves of glycogen, glucose homeostasis is finely regulated by insulin and counter-regulatory hormones: glucagon, epinephrine, cortisol and growth hormone (GH) [10,11] (Figure 1). After carbohydrate intake, plasma glucose levels increase and pancreatic beta-cells secrete insulin, which stimulates glucose uptake by peripheral tissues. Glucose is stored as glycogen in liver and skeletal muscles, and as triglycerides in adipose tissue. Insulin also inhibits glycogenolysis and hepatic gluconeogenesis. During fasting, insulin secretion is suppressed, while serum concentrations of counter-regulatory hormones increase. Thereby, glucose utilization by peripheral tissues is reduced; hepatic glycogenolysis is activated; and proteolysis and lipolysis are stimulated, providing liver substrates for gluconeogenesis. Ketone bodies are produced through fatty acid oxidation and they become the primary energy source for the brain. Particularly, when blood glucose concentration decreases below 80–85 mg/dL, insulin release is inhibited. If it drops further to 65–70 mg/dL, glucagon and epinephrine are secreted and they activate glycogenolysis. After 12–16 h of fasting, glycogen stores are depleted. If blood glucose falls below 65 mg/dL, cortisol and GH secretion increases, activating proteolysis, lipolysis and gluconeogenesis. Furthermore, epinephrine stimulates hepatic ketogenesis [11,12]. Infants and younger children have a relatively greater brain volume relative to body mass. They therefore exhibit a glucose utilization rate per kg of body weight 2–3 times higher than that of adults (4–6 mg/kg/min) [13].

### 1.2. Adaptation of Glucose Homeostasis from Intrauterine to Neonatal Life

During intrauterine life, the fetus receives glucose entirely through placental transfer. The acute interruption of this transfer at the time of delivery determines a quick reduction of blood glucose concentration during the first 2 h of life [14,15,16]. Insulin levels decrease to enable the mobilization of energy reserves, stored during intrauterine life as glycogen in the liver, proteins in the muscles and lipids in the adipose tissue. On the contrary, glucagon, epinephrine, cortisol and GH levels increase, activating glycogenolysis, gluconeogenesis and ketogenesis [17,18]. Plasma glucose concentration stabilizes at 45–80 mg/dL during the first 48 h of life. Then, it slowly rises during the next 3–4 days of life, reaching normal child and adult values [8,14]. Risk factors for neonatal hypoglycemia include the following: prematurity (gestational age < 37 weeks), fetal growth restriction (IUGR) or small for gestational age (SGA), post-maturity (gestational age > 42 weeks), large for gestational age (LGA), maternal diabetes, perinatal stress, admission to neonatal intensive care unit, maternal use of beta-adrenergic agents or hypoglycemic agents, family history of a genetic form of hypoglycemia, and congenital syndromes associated with hypoglycemia [19].

## 2. Etiology

The main causes of hypoglycemia can be classified into endocrine or metabolic etiology.

### 2.1. Hormonal Causes of Hypoglycemia (Table 1)

#### 2.1.1. Hyperinsulinism

Hyperinsulinism is a medical condition characterized by inappropriately high insulin secretion compared to plasma glucose concentration, leading to a continuous stimulation of hepatic glycogen synthesis and glucose uptake by skeletal muscles and adipose tissue [20,21]. Hyperinsulinism can be transient, congenital or syndromic. Transient hyperinsulinism is a common cause of severe hypoglycemia in newborns and it can be found in children of diabetic mothers, IUGR, SGA or it can be secondary to perinatal stress [22]. The disease course is usually self-limiting and it is defined by resolution prior to 6 months of age [23]. Hyperinsulinism in infants of diabetic mothers, when diabetes in poorly controlled, is caused by prolonged intrauterine exposure to high blood glucose levels resulting in increased fetal growth and LGA. Immediately after birth, these infants are at high risk for hypoglycemia but, usually, this condition resolves spontaneously in a few days until the beta-pancreatic cells adapt to the normoglycemic state [24]. In addition, gestational diabetes mellitus (GDM) is associated with long-term complications for offspring including childhood obesity and glucose intolerance. The molecular basis of offspring adiposity and glucose intolerance is expected to be correlated to genetic and epigenetic modifications owing to intrauterine hyperglycemia [25]. In some cases, newborns experience a prolonged state of transient hyperinsulinism. This disorder seems to occur primarily in SGA and in otherwise normal newborn infants who suffered asphyxia or other stress at the time of delivery [24]. A majority of infants with perinatal stress are diazoxide-responsive and this condition resolves spontaneously within the first 3 months to 6 months of life [26]. Congenital hyperinsulinism (CH) is the most common cause of persistent hypoglycemia in infants and children [20,27]. It is associated with several mutations including channelopathies, due to subunit mutations of beta-cell ATP-sensitive potassium channels (K-ATP channels), and metabolopathies, due to activating mutations of the enzymes glucokinase and glutamate dehydrogenase [28,29,30,31]. In basal conditions, the membrane potential of beta-pancreatic cells is around −65 mV, by the action of the K-ATP channels formed by SUR1 and KIR6.2 subunits, which allows a passage of K+ ions. Inside pancreatic beta-cells, glucose is phosphorylated and generates ATP. The consequent closure of the K-ATP channels results in a blockage of potassium entry into the cell with a gradual depolarization, opening of voltage-gated calcium channels and exocytosis of insulin granules [31]. CH is associated with over 12 different genetic loci and the most frequent are inactivating mutations of ABCC8 and KCNJ11 [32], which encode SUR1 and KIR6.2 subunits, with a consequent lack of channels on the beta-cell membrane or channels impaired function and dysregulated insulin secretion [33,34]. Other inactivating mutations include Hepatic Nuclear Factor 4 Alpha (HNF4A) and Hepatic Nuclear Factor 1 Alpha (HNF1A). Mutations in the pancreatic transcription factors HNF4A and HNF1A, cause diazoxide-responsive CH. These factors seem to be correlated to the regulation of K-ATP channels. Infants with HNF4A-CH are typically macrosomal and the hypoglycemia resolves within the first several years of life. Indeed, a progressive beta-cell failure occurs and results in a monogenic form of early onset diabetes (maturity-onset diabetes of the young (MODY1)). A similar clinical progression (from CH to diabetes) has been described in individuals with mutations in HNF1A, which results in MODY3 [26]. Activating mutations of glucokinase (GCK) and glutamate dehydrogenase (GDH) determine ATP overproduction, persistent closure of K-ATP channels and inappropriate insulin secretion. Mutations of GLUD1, which encodes GDH, cause the second most common form of CH and is known as hyperinsulinism/hyperammonemia syndrome, because GDH is involved in amino acid-stimulated insulin secretion. Mutations commonly occur de novo (70%) in an autosomal dominant manner [35]. CH could be classified into focal or diffuse forms and is characterized by increased insulin secretion by pancreatic beta-cells, causing hypoglycemia associated with low/normal levels of ketones and fatty acids, absence of metabolic acidosis, and positive glycemic response to glucagon. CH is correlated with a high risk of neurological damage and developmental delays because of severe hypoglycemia and lack of alternative fuels such as ketones [35,36]. Once the laboratory diagnosis of CH on a critical sample has been established, a genetic test should be performed and ABCC8/ KCNJ11 mutations must be investigated first [35]. Moreover, imaging examinations make it possible to distinguish focal and diffuse forms of CH. Fluorine 18 L-3, 4-dihydroxyphenyalanine positron emission tomography–computed tomography (18F-DOPA-PET-CT) has a sensitivity of 88% and a specificity of 94%, and is based on the selective uptake of L-DOPA by beta-cells and its conversion to dopamine by the enzyme DOPA decarboxylase. Therefore, imaging with 18F-DOPA-PET-CT should be performed in all patients believed to have focal forms of CH [37,38]. Treatment includes an emergency approach that aims to quickly restore normal blood glucose values, based on the administration of intravenous glucose solution or intravenous glucagon administration, and a long-term therapy that aims to prevent brain damage and promote normal development and growth of the child [39]. The main drug used for medical therapy is diazoxide. Diazoxide is also used in prolonged transient hyperinsulinism secondary to risk factors such as IUGR and perinatal asphyxia but is ineffective in diffuse forms of CH. Diazoxide acts by binding to the ABCC8 subunit of the K-ATP channels and determines the channel opening, resulting in membrane hyperpolarization and inhibition of insulin release. One of the main adverse effects of diazoxide is fluid retention; so, this therapy should be used with caution in children with hyperinsulinism who are receiving large volumes of fluids to maintain normoglycemia. Fluid restriction before starting diazoxide therapy is commonly practiced, along with concomitant use of a thiazide diuretic such as chlorothiazide, which also has a synergic action on K-ATP channels [40]. Octreotide represents a second-line treatment in cases of insensitiveness to diazoxide; it can be also used in combination with diazoxide in cases of partial response to diazoxide [41]. Octreotide binds to somatostatin receptors SSTR-2 and SSTR-5 and induces a hyperpolarization of the beta-cell membrane potential with blockage of calcium channels. Long-acting release (LAR) somatostatin analogs (lanreotide and LAR-octreotide) have the advantage of being administered monthly but they take a long time to reach a steady state; so, initially, they should be administered together with octreotide [42]. Potential new therapies are represented by glucagon-like peptide-1 receptor (GLP-1R) antagonists and pharmacological chaperones. Exendin 9–39 antagonizes the action of GLP-1, which is a hormone produced by the gut that stimulates insulin secretion and inhibits glucagon secretion by the pancreas [43,44]. Pharmacological chaperones are molecules that can affect channel biogenesis and trafficking defects. They facilitate protein folding and assembly by binding to their subunits. For example, it was recently observed that carbamazepine would act as a K-ATP channel chaperone [45,46]. Hyperinsulinism may occur also in the setting of specific syndromes, such as Beckwith–Wiedemann syndrome (BWS) [47,48]. BWS is a multisystem disorder caused by epigenetic or genomic alterations leading to abnormal methylation at a distinct differentially methylated region in 11p15.5 [49]. Hypoglycemia occurs in about 30–60% of patients and is related to excess insulin. Hyperinsulinism is usually transient and resolves within a few days, but persistent hyperinsulinism occurs in some cases. Hyperinsulinism may require medical treatment and usually is responsive to diazoxide. If medical treatment is not effective, surgical treatment may be used. In patients with focal forms, selective resection may be resolving; in diffuse forms requiring subtotal resection, pancreatic exocrine function replacement therapy and insulin therapy are obvious [50].

#### 2.1.2. Counter-Regulatory Hormone Defects

Counter-regulatory hormone deficiencies may be isolated or part of panhypopituitarism. Hypopituitarism should be suspected in children with midline defects (such as cleft lip or cleft palatal), optic nerve hypoplasia and, in males, micropenis [51]. Isolated GH deficiency (GHD) is caused by mutations in the GHRH, GH or their respective receptors. GHD associated with hypopituitarism can be due to central nervous system abnormalities (such as empty sella syndrome, septo-optic dysplasia, pituitary hypoplasia/aplasia) or to acquired causes (such as meningitis, hydrocephalus, radiotherapy, head injury, cerebral infarction, cancer, and surgery) [52,53]. GHD leads to decreased lipolysis and glycogenolysis [54]. Metabolic effects of GHD are represented by ketotic hypoglycemia associated with fasting metabolic acidosis [55]. However, in infants, it presents with a clinical picture identical to that of hyperinsulinism (hypoglycemia associated with low/normal levels of ketone bodies and fatty acids, absence of metabolic acidosis and positive response to glucagon administration). Other clinical features are prolonged and recurrent jaundice in infants, early and progressive growth deceleration during the first years of life, and dysmorphic features with midline defects (in hypopituitarism) occur [56]. The treatment is hormone-replacement therapy; in cases of persistent hypoglycemia, GH treatment can be initiated during the neonatal period with daily subcutaneous injections of recombinant human GH in the evening to mimic the physiological release of GH. The recommended starting dose is 22–35 mcg/kg per day. Lower doses (10–20 mcg/kg/day) can also lead to excellent responses in this age group. Dose adjustments are recommended every 6–12 months based on growth response [57]. Cortisol deficiency can be divided into primary adrenal insufficiency and secondary adrenal insufficiency. Primary adrenal insufficiency may be due to congenital adrenal hyperplasia or to acquired causes (such as Addison’s disease, adrenal hemorrhage, adrenal infarction, infections, and drugs). Secondary adrenal insufficiency is most often due to prolonged steroid therapies and rarely to congenital causes (CRH deficiency, ACTH deficiency, and hypopituitarism) [58]. Cortisol deficiency leads to decreased proteolysis, lipolysis and gluconeogenesis, resulting in an incapacity to raise glucose levels [59,60,61]. Cortisol deficiency causes hypoglycemia during fasting, especially in stressful conditions, with increased ketone bodies and metabolic acidosis. As in GHD, the biochemical features in infants are identical to those of hyperinsulinism (hypoglycemia associated with low/normal levels of ketone bodies and fatty acids, absence of metabolic acidosis and positive response to glucagon administration). Moreover, cortisol-deficient infants may present with cholestasis during the first 2 weeks of life [62,63]. Indeed, cortisol increases bile flow and, therefore, its deficiency will cause abnormalities in bile acid synthesis and transport leading to cholestasis in some cases. Hydrocortisone represents the best treatment; the daily dose of hydrocortisone varies between 7.5–15 mg/m^2^/day, administered in 3–4 doses. In the event of illness or stress, hydrocortisone doses should be doubled or even tripled [57,64].

#### 2.1.3. Other Endocrine Causes: Insulin-Like Growth Factor-II (IGF-II) Production

Insulin-like growth factor II (IGF-II) is a protein structurally similar to insulin which represents the major insulin-like growth factor during fetal life. It exerts its action through insulin receptors (IR) and/or IGF-1 receptors (IGF-1R) [65]. Some neoplastic formations most commonly found in children (Wilms’ tumor, nephroblastoma, dysgerminomas, and lymphomas/leukemias) are associated with increased IGF-II secretion [66]. The continued activation of insulin-related receptors by IGF-II leads to uptake of glucose mainly by skeletal muscle, suppression of free fatty acid release by adipocytes, and the inhibition of glucose release, glycogenolysis, gluconeogenesis, and ketogenesis in the liver. This leads to fasting hypoglycemia with low ketone levels in the absence of elevated insulin and C-peptide values [67]. In addition, both glucagon and GH release are suppressed by IGF-II: circulating glucagon is suppressed due to the dual action of IGF-II on IGF-1R and IR of pancreatic alpha cells; the low level of GH is probably due to the negative feedback of IGF-II via IGF-1R in the cells of the hypothalamus [68]. High levels of IGF-II also suppress normal insulin secretion by pancreatic beta-cells, which may lead to postprandial hyperglycemia in the context of fasting hypoglycemia. Medical treatment is the administration of glucocorticoids and GH; new therapies are anti-IGF-II monoclonal anti-bodies. However, the gold standard of treatment of IGF-II-oma hypoglycemia remains surgical resection [69].

**Table 1:** Main endocrinologic causes of hypoglycemia. Abbreviations: M.A.: metabolic acidosis. The up arrow indicates an increased synthesis or action of the respective element. The down arrow indicates a deficiency in synthesis or action of the respective element.

	Type	Causes	Clinical Features	Biochemical Features	Treatment	Potential New Therapies
	Hyperinsulinism	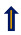 Insulin	Hypoglycemic symptoms	Hypoglycemia, increased insulin and C-peptide, normal ketones and fatty acids, absence of M.A., positive glycemic response to glucagon	Diazoxide, somatostatin analogs (octreotide), long-acting release somatostatin analogs (lanreotide)	Glucagon-like peptide-1 receptor antagonists, pharmacological chaperones
	GH deficiency	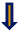 GH	Hypoglycemic symptoms, prolonged and recurrent jaundice, growth deceleration	Hypoglycemia, increased ketones, fasting M.A.	Hormone replacement therapy	
Endocrine disorders	Adrenal insufficiency	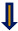 Cortisol	Hypoglycemic symptoms, cholestasis	Fasting hypoglycemia, increased ketones, fasting M.A.	Hormone replacement therapy	
	Pediatric neoplastic formations (Wilms’ tumor, nephroblastoma, lymphomas/leukemias)	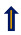 IGF-II	Hypoglycemic symptoms	Fasting hypoglycemia and postprandial hyperglycemia, suppressed insulin secretion, low ketones and fatty acids, suppressed glucagon and GH release	Surgical treatment, glucocorticoids and GH administration	Anti-IGF-II monoclonal antibodies

### 2.2. Metabolic Causes of Hypoglycemia, (Table 2 and Figure 2)

#### 2.2.1. Glycogen Storage Disorders

Glycogen storage disorders (GSDs), also named glycogenosis, represent a group of inherited metabolic diseases caused by abnormalities or deficits in enzymes involved in glycogen synthesis and degradation. Glycogen represents the main reserve of glucose and it is mainly stored in the liver, muscles, and kidneys. Hypoglycemia is the primary manifestation of liver GSD (types 0, I, III, VI, IX, and XI), while weakness and/or muscle cramps are the primary features of muscle GSD (types II, III, IV, V, VII, X). Type III is characterized by concomitant liver and muscle involvement. Type IV (branched enzyme deficiency) causes liver fibrosis and cirrhosis in its classic form; in this case, hypoglycemia occurs when liver failure takes over [70,71]. Glycogenosis type 0 is caused by a deficiency in glycogen synthase, leading to a marked decrease in hepatic glycogen content. After carbohydrate consumption, the inability to store glucose as glycogen in the liver results in postprandial hyperglycemia and hyperlactatemia. Fasting can cause severe ketotic hypoglycemia. Postprandial hyperglycemia and fasting ketonuria may be confused the onset of type 1 diabetes (T1D) [72]. Glycogenosis type Ia and Ib (von Gierke disease) account for over 80% of GSD cases and result, respectively from a deficiency of glucose-6-phosphatase (G6P) and glucose 6-phosphate transporter deficiency. Hydrolysis of glucose 6-phosphate to glucose, the final reaction of both glycogenolysis and gluconeogenesis, is impaired [73]. GSD I is associated with the most severe fasting intolerance. Plasma lactate starts to increase when blood glucose falls below 70 mg/dL (3.9 mmol/L). Glycogen accumulation causes hepatomegaly and nephromegaly and failure to thrive, and G6P deficiency results in alternative metabolic pathways leading to hyperlactatemia, hyperuricemia (pentose-phosphate pathway), and hypertriglyceridemia. Unlike other glycogenoses, GSD-I is associated with hypoketotic hypoglycemia (fatty acids liberated from lipolysis are esterified with glyceraldehyde 3-phosphate, which accumulates as an intermediate metabolite, thus being diverted from the process of ketogenesis). Almost all patients with GSD Ia have manifestations in the neonatal period, but the diagnosis is rarely made because the clinical manifestations become less obvious with the onset of frequent feedings. Later, when the interval between feedings becomes longer, the first manifestations begin [74]. Type Ib glycogenosis is clinically identical to type Ia glycogenosis. With advancing age, however, most patients develop neutropenia, neutrophil dysfunction and inflammatory bowel disease (IBD) [75]. Glycogenosis type III (Cori or Forbes disease) is characterized by glycogenolysis arrest and glycogen accumulation in affected tissues. Hepatic involvement leads to hepatomegaly and fasting hypoglycemia. Patients with GSD III can synthesize glucose by gluconeogenesis, and energy formation from fatty acid oxidation is possible [76]. Consequently, hypoglycemia is often not as severe as in GSD I and is typically associated with ketosis and hyperlipidemia. Muscle involvement leads to chronic myopathy, muscle weakness, and pain, but these manifestations do not typically occur in childhood [77]. Glycogenosis type VI (Hers disease) and Glycogenosis type IX are caused, respectively, by hepatic glycogen phosphorylase and glycogen phosphorylase kinase deficiencies. Since phosphorylase kinase is required to activate glycogen phosphorylase, GSD VI and IX show a similar clinical presentation. Usually, patients present growth retardation, hypotonia, and hepatomegaly. Ketotic hypoglycemia may occur. However, hypoglycemia is often not recognized because ketones attenuate neuroglycopenic symptoms [78]. Glycogenosis maintenance treatment is represented by a dietary approach (use complex sugars such as cornstarch, that allow prolonged carbohydrate release; protein supplementation as substrate for endogenous glucose production) in order to reduce fasting time and maintain constant glucose concentrations (above 75 mg/dL and below 100 mg/dL) to minimize glycogen accumulation [79]. The lack of specific therapy for GSD has prompted the development of new therapies for these conditions. New therapeutic prospects for GSDs are represented by gene therapy. Gene therapy aims to replace deficient enzymes in target tissues by viral vectors that can reach the target tissue. Gene therapy has been advanced to the initial phase of clinical trials for the replacement of G6P in GSD Ia and acid α-glucosidase (GAA) in GSD II (Pompe disease) [72,80].

**Table 2:** Main metabolic causes of hypoglycemia. Abbreviations: GSD: glycogen storage disease, HFI: hereditary fructose intolerance, FAO: fatty acid oxidation. The up arrow indicates an increased synthesis or action of the respective element. The down arrow indicates a deficiency in synthesis or action of the respective element.

	Type	Causes	Clinical Features	Biochemical Features	Treatment	Potential New Therapies
	GSD 0	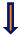 Glycogen synthase	Hypoglycemic symptoms, normal liver size	Postprandial hyperglycemia and hyperlactatemia, fasting ketotic hypoglycemia	Dietary approach	
Metabolic disorders	GSD Ia	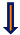 Glucose-6- phosphatase	Hypoglycemic symptoms, hepatomegaly, nephromegaly, failure to thrive	Fasting hypoglycemia, mild ketosis, hyperlactatemia, hyperuricemia, hypertriglyceridemia	Dietary approach	Gene therapy (initial phase of clinical trials)
	GSD Ib	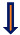 Glucose-6-phosphatase transporter	Hypoglycemic symptoms, hepatomegaly, nephromegaly, failure to thrive, neutropenia, inflammatory bowel disease	Fasting hypoglycemia, mild ketosis, hyperlactatemia, hyperuricemia, hypertriglyceridemia	Dietary approach	
	GSD III	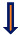 Glycogen debranching enzyme	Hypoglycemic symptoms, hepatomegaly, chronic myopathy, delayed growth	Fasting ketotic hypoglycemia, hyperlipidemia, increased CPK and transaminases	Dietary approach	
	GSD VI, IX	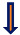 Glycogen phosphorylase, Phosphorylase kinase	Hypoglycemic symptoms, hepatomegaly, failure to thrive, hypotonia	Fasting ketotic hypoglycemia, mild hyperlipidemia	Dietary approach	
	GSD XI	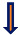 GLUT2 transporter	Hypoglycemic symptoms, hepatomegaly, failure to thrive, Fanconi tubulopaty	Fasting hypoglycemia, hyperaminoaciduria, acidosis, hyperphosphaturia, glycosuria	Dietary approach	
	HFI	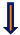 Aldolase B	Acute signs and symptoms (nausea, vomiting, abdominal pain, lethargy, seizures) and chronic signs (failure to thrive, hepatic and renal insufficiency)	Postprandial hypoglycemia, lactic acidemia, hypophosphatemia, hyperuricemia, hypermagnesemia, hyperalaninemia	Dietary restriction of fructose, sucrose, sucralose, and sorbitol	
	Galactosemia	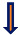 GALT, GALK, GALE	Hypoglicemic symptoms, poor feeding, vomiting, jaundice, hepatomegaly, hypotonia, lethargy, cataracts, ovarian failure, failure to thrive	Postprandial hypoglycemia, hyperchloremic metabolic acidosis, hypophosphatemia, increased transaminases and direct/indirect bilirubin, aminoaciduria	Galactose restricted diet	Gene therapy, pharmacological chaperones, enzyme inhibitors, endoplasmic reticulum stress-reducing agents
	Gluconeogenesis disorders	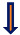 Fructose-1,6-bisphosphatase	Hypoglycemic symptoms, hepatomegaly, delayed growth	Fasting ketotic hypoglycemia, lactic acidosis, hyperuricemia, hypertriglyceridemia	Dietary approach	
		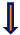 Pyruvate carboxylase	Hypoglycemic symptoms, severe encephalopathy, developmental delay, seizures, growth retardation	Fasting ketotic hypoglycemia, metabolic acidosis	Dietary approach	
		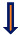 Phosphoenolpyruvate carboxykinase	Hypoglycemic symptoms	Fasting ketotic hypoglycemia, metabolic acidosis	Dietary approach	
	Fatty-acid-oxidation disorders	Defects of enzymes involved in transport and beta oxidation of F.A. in the mitochondria	Hypoglycemic symptoms, cardiomyopathy, myopathy, hepatomegaly, Reye-like syndrome	Hypoketotic hypoglycemia, elevated free fatty acids, increased transaminases and CPK, hyperammonemia	Dietary approach	Triheptanoin for long-chain FAO disorders

**Figure 2:**
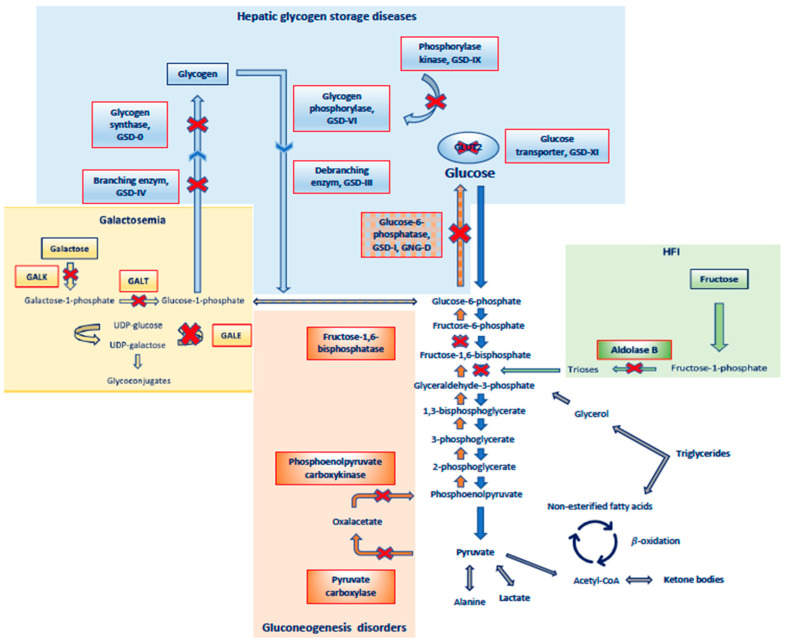
Major disorders of carbohydrate metabolism related to hypoglycemia. Abbreviations: GSD: glycogen storage disease, GNG-D: gluconeogenesis disorder, HFI: hereditary fructose intolerance.

#### 2.2.2. Hereditary Fructose Intolerance

Hereditary fructose intolerance (HFI) is caused by loss of function of the enzyme aldolase B (whose gene is located on chromosome 9q22.3) with autosomal recessive inheritance. Aldolase B is involved in fructose metabolism and performs its function mainly in the liver, kidney cortical and intestinal mucosa [81]. Accumulation of fructose-1-phosphate due to reduced function of aldolase B inhibits gluconeogenesis and glycogenolysis. Although HFI typically first manifests with the introduction of fructose- and sucrose-containing foods during weaning, it may occur earlier due to the addition of fructose-containing nutrients in infant formulae. Acute signs and symptoms are nausea, vomiting, abdominal pain, lethargy, convulsions, and/or progressive coma; impaired gluconeogenesis following fructose ingestion causes acute hypoglycemia refractory to glucagon [82]. HFI is one of the few congenital errors of metabolism in which hypoglycemia occurs in the immediate postprandial state. Chronic fructose exposure causes growth retardation, and liver and kidney failure. Management of acute events includes intravenous administration of glucose, supportive treatment of hepatic and/or renal failure and treatment of metabolic acidosis [83]. Long-term management provides dietary restriction of fructose, sucrose, and sorbitol; daily supplementation with a “sugar-free” multivitamin is recommended to prevent micronutrient deficiencies, especially water-soluble vitamins; periodic evaluations of liver and kidney functionality and growth are also recommended. Although vaccinations are generally safe in children with HFI, the two potentially dangerous vaccines are the sucrose-containing rotavirus vaccines, Rotarix^®^ and RotaTeq^®^. These are routinely administered at ages two months, four months, and six months, usually before a child is found to have HFI. Therefore, any child with vomiting, hypoglycemia, lethargy, or unexplained liver or kidney failure after rotavirus vaccination should be thoroughly investigated for the possibility of HFI [84].

#### 2.2.3. Galactosemia

Hereditary galactosemia is among the most relevant disorders of carbohydrate metabolism [81]. Galactose is metabolized by the enzymes of the Leloir pathway: galactokinase (GALK), galactose-1-phosphate uridyl-transferase (GALT, whose gene is located on chromosome 9p13) and UDP-galactose 4-epimerase (GALE). Deficiency in either of these enzymes with autosomal recessive transmission causes galactosemia. There are three types of galactosemia: classic or type I galactosemia (GALT deficiency), type II galactosemia (GALK deficiency) and galactosemia type III (GALE deficiency) [85,86]. Deficiency of an enzyme of the Leloir pathway causes the accumulation of intermediate metabolites, namely galactitol and galactonate. The intracellular increase in galactitol induces cellular oxidative stress, which is the etiologic agent of cataracts in GALT and GALK deficiencies. The most common is type I or classic galactosemia [87]. The diagnosis of classic galactosemia can be formulated by the patient’s clinical features, neonatal screening results and/or laboratory findings. Although, many countries have introduced galactosemia into their neonatal screening programs, it has still not spread universally throughout Europe, mainly due to the high rate of false-positive results. The diagnosis is established by measuring galactose-1-phosphate levels and GALT enzyme activity in red blood cells and/or by genetic testing of the GALT gene. Regarding laboratory data, an increase in serum indirect and/or direct bilirubin, increase in transaminases, and decrease in fibrinogen may be found. If undiagnosed, patients may also present prolonged PT and PTT, hypoglycemia, hyperchloremic metabolic acidosis, hypophosphatemia, aminoaciduria, and decreased hemoglobin/hematocrit [88]. The treatment of classic galactosemia is a lifelong galactose-free diet. Although implementation of a low-galactose diet is effective in resolving acute complications, it is not sufficient to prevent long-term complications affecting the central nervous system and the female gonads, the two main target organs. Galactosemia type II results from GALK deficiency. The main clinical feature is cataracts in the neonatal period, which can be resolved or prevented by a galactose-reduced diet. Cataract formation appears to be attributed to galactitol accumulation in the lens. Type III galactosemia results from reduced GALE activity. The generalized form represents the most severe form, in which there is a GALE deficiency in all tissues. It presents with acute clinical symptoms, similar to those of GALT deficiency, including hypotonia, vomiting, weight loss, jaundice, hepatomegaly, and liver failure. Long-term complications of a generalized form may include cognitive impairment, developmental delay, and poor growth [89]. Currently, preclinical and/or clinical studies are being conducted in order to identify new therapies with the aim to prevent long-term complications. These include gene therapy via adenovirus viral vectors and mRNA therapy that, via various vehicles (e.g., liposomes, nanoparticles, and viruses) reaches the target tissue and is translated into a functional protein. Pharmacological chaperones, enzyme inhibitors, and endoplasmic reticulum (ER) stress-reducing agents should also be mentioned. Enzyme inhibitors include galactokinase 1 (GALK1) inhibitors and aldose reductase (AR) inhibitors. GALK1 inhibitors aim at reducing galactose 1-P accumulation, which plays a key role in the pathogenesis of CG. Among these new therapies, the most promising would seem to be gene therapy and enzyme inhibitors; however, further studies are needed to demonstrate the actual efficacy of these techniques [90].

#### 2.2.4. Gluconeogenesis Disorders

Gluconeogenesis (GNG) is a metabolic process characterized by the conversion of substrates such as lactate, pyruvate, alanine and glycerol into glucose, in order to maintain normal blood glucose levels during fasting. Conversion of pyruvate to glucose is the central pathway for GNG reactions. The glycolysis and GNG pathways are nearly identical, and defects of multiple enzymes, such as glucose-6-phosphatase, fructose-1,6-bisphosphatase, pyruvate carboxylase (PC), and phosphoenolpyruvate carboxykinase, can cause GNG disorders [91]. Hepatic glucose production is impaired and the accumulation of gluconeogenic substrates in the liver results in significant metabolic abnormalities [92]. Among the most frequent enzyme deficits, there are fructose-1,6-bisphosphatase and pyruvate carboxylase deficiencies. Fructose-1,6-bisphosphatase catalyzes the hydrolysis of fructose 1,6-bisphosphate to fructose 6-phosphate. Its deficiency is associated with fasting hypoglycemia, severe lactic acidosis usually with ketosis, hyperuricemia, hypertriglyceridemia, growth retardation, and moderate hepatomegaly. This condition might be fatal during the neonatal period due to severe hypoglycemia and respiratory distress associated with metabolic acidosis. The diagnosis is suspected through biochemical features and is confirmed by genetic testing of the FBP1 gene. Treatment includes frequent feedings and avoidance of prolonged fasting, and prognosis is good with proper dietary management [93,94]. PC deficiency is a defect involving both gluconeogenesis and the Krebs cycle. This disorder usually presents with fasting hypoglycemia, severe encephalopathy, developmental delay, seizures, growth retardation, and metabolic acidosis. Treatment includes an intravenous infusion of glucose, metabolic acidosis correction and dietary management [95,96].

#### 2.2.5. Fatty-Acid-Oxidation Disorders

Mitochondrial fatty-acid-oxidation (mFAO) disorders are due to genetic defects of enzymes involved in their transport and beta oxidation inside of the mitochondria, resulting in a lack of substrates for ketogenesis [97,98]. The most common is medium-chain acyl-CoA dehydrogenase deficiency. Fatty acids are used as an energy substrate preferentially by skeletal muscles and particularly by cardiac muscle. During prolonged fasting, most organs utilize fatty acids in order to save glucose for the brain. The release of fatty acids for beta-oxidation is stimulated by epinephrine, norepinephrine, glucagon and adrenocorticotropic hormone (ACTH) in response to fasting, stimulating lipolysis in adipose tissue [99]. Hereditary disorders of fatty acid metabolism may involve three main processes: the carnitine uptake system on the plasma membrane through which fatty acid molecules are transported within the cell; the carnitine shuttle system that allows the transport of long-chain acyl-CoA into the mitochondria (medium- and short-chain fatty acids are able to enter the mitochondrial matrix independently of carnitine); beta-oxidation of fatty acids, which is the main pathway of fatty acid degradation [100]. Clinically, fatty acid metabolism disorders are characterized by hypoketotic hypoglycemia, often accompanied by elevated transaminase levels, hepatomegaly and liver failure associated with elevated fatty acid levels. In some cases, there is an acute onset with Reye’s syndrome (acute liver failure and encephalopathy) and sudden unexpected infant death. Moreover, these disorders are characterized by cardiac involvement with arrhythmias and myopathy with elevated creatinine kinase levels, muscle weakness, or recurrent rhabdomyolysis in adolescence or adulthood [101,102]. In these children, a prolonged fasting of 12–18 h can lead to acute metabolic decompensation. The mortality rate can be as high as 25%. These episodes occur in infancy or early childhood, when the children sleep longer without feeding, or during an intercurrent illness that compromises oral feeding [97]. Fortunately, these metabolic disorders are being detected early through extended newborn screening [103]. Treatment consists of avoiding prolonged fasting, ensuring adequate nutritional intake through a carbohydrate-rich diet to maintain euglycemia, and resorting to carnitine supplementation if deficient. Regarding long-chain-fatty-acid-oxidation disorders (LC-FAO), triheptanoin, a heptanoyl–triglyceride dietary supplement, was approved by the FDA in June 2020 as a new therapy. Upon ingestion, triheptanoin is rapidly digested into heptanoate in the small intestine and absorbed into the circulation [104]. Like other medium-chain fats, heptanoate can diffuse through the mitochondrial membrane and enter the fatty-acid-oxidation cycle, circumventing the need for the carnitine cycle. Clinical studies conducted have shown improvement in clinical symptoms [98].

## 3. Idiopathic Ketotic Hypoglycemia

Idiopathic ketotic hypoglycemia (IKH) is the most frequent form of hypoglycemia in healthy children aged 18 months to 7 years. It spontaneously resolves around 9 years of age, when children present an increase in their lean mass and a decrease in their glucose requirement per unit of body mass [105,106]. IKH is an exclusion diagnosis. It usually occurs in the morning after fasting overnight or during an intercurrent illness, due to an increased metabolism. It is likely be due to a lower endogenous production of glucose, secondary to a reduced availability of amino acids for gluconeogenesis. It is more evident in younger children because they present limited glycogen stores and high glucose utilization [107]. PES recommends maintaining plasma glucose levels > 70 mg/dl and beta-hydroxybutyrate (BOHB) < 1 mmol/L through nutritional therapy and avoiding prolonged fasting or increasing frequency of feeding, especially during stress or illness episodes [108].

## 4. Conclusions

Hypoglycemia represents a common metabolic-endocrine emergency that might cause permanent neurological consequences, if not treated promptly. However, especially in children, hypoglycemia might be difficult to recognize because the signs and symptoms are nonspecific, and very often children are unable to communicate them. The causes of hypoglycemia are numerous and may recognize an endocrine or metabolic etiology. Over the past few years, much progress has been made in both the early diagnosis and treatment of these conditions. Extended newborn screening allows the early detection of many metabolic disorders at birth and new therapies are being studied which might improve the outcome and quality of life of these patients. Although much progress has been made, further studies are needed in order to improve the diagnoses and introduce these new therapeutic strategies into clinical practice.

## Data Availability

Not applicable.
